# Metacognitive Training for Obsessive-Compulsive Disorder: a study protocol for a randomized controlled trial

**DOI:** 10.1186/s12888-020-02648-3

**Published:** 2020-07-06

**Authors:** Franziska Miegel, Cüneyt Demiralay, Steffen Moritz, Janina Wirtz, Birgit Hottenrott, Lena Jelinek

**Affiliations:** Department of Psychiatry and Psychotherapy, University Medical Center Hamburg-Eppendorf, Martinistrasse 52, 20246 Hamburg, Germany

**Keywords:** Beliefs, Group therapy, CBT, Anxiety, Metacognitions, Biases

## Abstract

**Background:**

A high number of patients with obsessive-compulsive disorder (OCD) do not receive cognitive-behavioral therapy with exposure and response prevention, which is the most effective treatment for OCD. Therefore, Metacognitive Training for OCD (MCT-OCD) was developed, which is a structured group therapy aiming at the modification of dysfunctional (meta-)cognitive biases, beliefs and coping styles. It can be administered by less trained personnel, thus may reach a higher number of patients.

An uncontrolled pilot study (MCT-OCD pilot version) provided first evidence that the training is highly accepted by patients; OC symptoms decreased with a high effect size (η^2^_partial_ = 0.50). The aim of the present study is to address the shortcomings of the pilot study (e.g., no control group) and to assess the efficacy of the revised version of the MCT-OCD in the framework of a randomized controlled trial.

**Methods:**

Eighty patients with OCD will be recruited. After a blinded assessment at baseline (−t1), patients will be randomly assigned either to the intervention group (MCT-OCD; *n* = 40) or to a care as usual control group (*n* = 40). The MCT-OCD aims to enhance patients’ metacognitive competence in eight modules by addressing dysfunctional (meta-)cognitive biases and beliefs associated with OCD (e.g., intolerance of uncertainty). After 8 weeks, patients will be invited to a post assessment (t1), and then they will receive a follow-up online questionnaire 3 months following t1 (t2). The primary outcome is the Y-BOCS total score, and the secondary outcomes include the HDRS, OCI-R, OBQ-44, MCQ-30, WHOQOL-BREF, BDI-II, and subjective appraisal ratings of the MCT-OCD. We expect that OC symptoms will decrease more in the intervention group compared with the care as usual control group from –t1 to t1 and that treatment gains will be maintained until t2.

**Discussion:**

The planned study is the first to investigate the MCT-OCD, a promising new treatment, in a randomized controlled trial. The MCT-OCD may help to overcome existing treatment barriers for patients with OCD.

**Trial registration:**

German Registry for Clinical Studies (DRKS00013539), 22.02.2018.

## Background

Obsessive-compulsive disorder (OCD) is characterized by intrusive, repetitive, and perturbing thoughts (i.e., obsessions such as the fear of being infected by germs when touching a door handle) that usually evoke negative feelings (e.g., fear or disgust) [1]. These negative emotions are reduced or occur less frequently, respectively upon execution of compulsive behavior that is ritualized and repetitive (e.g., excessive hand washing) and of avoidance behavior (e.g., pushing the door handle down with one’s elbow), but these behaviors maintain OC symptoms over the long term. OCD has a lifetime prevalence of 2–3% [2] and often has a chronic course [3]. Quality of life in patients with OCD is usually low [4, 5], even following clinically successful treatment.

### Maintaining factors of OCD

According to the cognitive model of OCD [6–8], emotional processing theory [9], and Wells’ metacognitive model [10–12], dysfunctional beliefs play an important role in the development as well as maintenance of OCD. For heuristic purposes and in order to classify the constructs into the different elements of the metacognitive training for OCD (MCT-OCD), which is described below, we think it is necessary to distinguish between beliefs, metacognitive beliefs, cognitive biases, and coping strategies. In the following, we describe our understanding of how the terms can be distinguished from each other. A belief may be defined as “an enduring organization of perceptions and cognitions about some aspect of the individual’s world” ([13], p. 152). Metacognitive beliefs^1^ are beliefs that are concerned with cognitive processes, as, for example, thought-action fusion (TAF; i.e., the belief that thoughts equal a person’s actions or that they may be followed by moral consequences) [10, 11]. Cognitive biases are “distortions in the way an individual perceives, interprets and recollects information” ([15], p. 4) and are automatic (not conscious) rather than controlled. The most common definition of coping strategies is that of Lazarus and Folkman [16]: “Constantly changing cognitive and behavioral efforts to manage specific external and/or internal demands that are appraised as taxing or exceeding the resources of the person” (p. 141). Often, however, the boundaries of these concepts (beliefs, metacognitive beliefs, cognitive biases, and coping strategies) are blurred.

Many studies show that dysfunctional (meta-)cognitive beliefs, cognitive biases and coping strategies are associated with OC symptoms and that targeting these features may reduce OC symptoms. For example, investigating mechanisms of change in a cognitive therapy (CT) for patients with OCD indicated that the amelioration of beliefs mediated treatment success [17]. Another study showed that the need to control thoughts during exposure therapy predicted a subsequent improvement in OC symptoms [18].

### Psychological treatments for patients with OCD

Cognitive-behavioral therapy (CBT) with exposure and response prevention (ERP) as well as pharmacotherapy (i.e., selective serotonin reuptake inhibitors) are evidence-based treatments for OCD [19]. Because the risk-to-benefit ratio and the acceptability by patients is better for CBT compared to pharmacotherapy, CBT is usually recommended as first-line treatment [20]. CBT (“either described as such or as the combination of ERP and cognitive therapy”) ([95], p. 159) has been found to be superior to wait-list controls in patients with OCD, with a large effect size (Hedges’s *g* = 1.31) [95]. On the one hand, 75–80% of patients with OCD respond to ERP (reduction of ≥35% of the Y-BOCS score), but, on the other hand, only 40–73% achieve remission (depending on the definition of a Y-BOCS score ≤ 14 or ≤ 12) [21–23]. Moreover, the dropout rate for ERP is quite high (14.7%) [96] and therapists often avoid offering ERP, so that 40% of OCD patients do not receive CBT with ERP [24, 25].

CBT delivered in a group setting is generally seen as a good and cost-effective alternative or supplement to individual CBT. Although some studies have found that individual CBT is superior to group CBT in patients with OCD [26, 27], most studies report that both are equally effective [29, 97]. Interestingly, group CBT has been shown to be effective over a long period of time (i.e., at 3- and 12-month follow-ups) [28]. Another meta-analysis [29] confirmed that group therapy is highly efficacious in reducing OC symptoms compared to wait-list control groups (*g* = 0.97, 95% CI 0.58; 1.37, *p* < 0.001, *k* = 4). Additionally, Schwartze et al. [29] determined that group therapy is similar in effectiveness compared to individual therapy or pharmacotherapy. The authors emphasize that more research is needed in order to evaluate group therapy approaches other than CBT.

CT, which in contrast to CBT focuses on the cognitive elements and does not include ERP [30], is recommended by the guidelines of the National Institute for Health and Care Excellence (NICE) [20] and is also frequently chosen as a treatment for OCD. The aim of CT is to modify dysfunctional interpretations of obsessions by establishing more helpful interpretations [31]. A recent study by Steketee, Siev, Yovel, Lit, and Wilhelm [32] as well as two meta-analyses showed that CT and ERP both reduced OC symptoms to a similar extent [33, 34]. A treatment approach that builds upon the metacognitive model [10] and focuses on dysfunctional metacognitive beliefs (e.g., TAF) is the metacognitive therapy developed by Wells [35]. This treatment does not aim at modifying dysfunctional interpretations of obsessions – as in CT – but rather focuses on metacognitive beliefs [35], that is, on thinking *processes* instead of content. Moreover, behavioral experiments are part of the intervention, but ERP is not [36]. Two studies suggest that Wells’ metacognitive therapy is effective in OCD (decreases in Y-BOCS from baseline to post: *d* = 2.28, *d* = 2.54) [35, 36]. However, these studies lacked a control group, limiting the conclusions. Using a non-randomized-design, Wells’ metacognitive therapy was compared to group CBT in OCD, and the metacognitive therapy showed a superior response rate (a 86.3% response rate for metacognitive therapy compared to a 64% response rate for CBT) [37]. However, the study was limited by a lack of control for pharmacotherapy, a lack of clinician-administered interviews (apart from the Structured Clinical Interview for DSM-IV), and untreated control conditions.

### Treatment barriers for patients with OCD

Although the above treatments are effective for many patients, various factors compromise their dissemination and acceptance. For example, Mancebo, Eisen, Sibrava, Dyck, and Rasmussen [38] found that one-quarter of patients with OCD who received the recommendation to start CBT with ERP did not follow the suggestion. Voderholzer et al. [24] suggest that the fear of being confronted with anxiety-inducing stimuli might be one of the reasons why patients do not want to undergo this treatment. Additionally, CBT, CT, and the metacognitive therapy developed by Wells need to be conducted by a trained professional. As training is time-consuming and costly, trained professionals are few in number and are not always available, especially outside urban areas, which result in a high number of patients remaining untreated. In addition, in Germany, for example, patients wait on average 5 months for psychotherapy despite the country’s advanced mental health network [39]. Therefore, it is important to introduce new treatments to mental health care that are available to more patients, can be administered by therapists with less training, and do not need a lot of time for preparation and thus can be easily disseminated.

### Metacognitive training for patients with OCD

In order to address some of the aforementioned treatment barriers, our working group developed a group therapy for patients with OCD, Metacognitive Training for OCD (MCT-OCD), which is derived from our Metacognitive Training for psychosis (MCT) [98]. Two meta-analyses of MCT showed a moderate postintervention effect of *g* = − 0.34 [41] and *g* = − 0.38 [40]. The MCT for psychosis is the basis of other metacognitive trainings we developed, such as for depression [42] and borderline personality disorder [43]. Although the MCT-OCD has many overlaps with CT and CBT in terms of content, the focus of the interventions differs in that the MCT-OCD is more about sowing doubt regarding dysfunctional cognitive beliefs and biases than questioning dysfunctional assumptions or exposing patients to particular stimuli. In addition, the way the MCT-OCD is presented is quite different from CBT in that it is a slide-supported presentation that includes humorous exercises in order to provide corrective "aha moments," (violation of expectancy) thereby also aiming to enhance the awareness of dysfunctional mental processes in a normalizing and nonstigmatizing fashion (see Moritz and Lysaker [15] for a description of metacognitive aspects in MCT and other metacognitive interventions). Moreover, the overarching idea of metacognitive training is the modification of disorder-specific (meta-)cognitive beliefs, biases and coping strategies. The metacognitive trainings follow an open group format (i.e., all patients complete all modules but start with a different module) that allows patients to join the group at any time, which prevents long waiting times. The metacognitive trainings are highly standardized, thus less time is needed for preparation and administration, allowing for their easy dissemination and, consequently, facilitating treatment access for patients.

The present approach unites the general features of the MCTs (e.g., open group concept, inclusion of “aha moments”) with the contents of a self-help manual for patients suffering from OCD called “myMCT” (for “my metacognitive training”) developed by Moritz, Jelinek, Hauschildt, and Naber [44]. The manual includes psycho-education about core elements of OCD (i.e., obsessions, compulsions, avoidance, and safety behaviors), offers patients support in identifying dysfunctional (meta-)cognitive biases as well as dysfunctional coping strategies, and provides new strategies. The myMCT has already shown to be superior to wait-list as well as active (psycho-education) control groups over a period of 4 weeks [44–46]. A recent meta-analysis showed an effect size of *SMD* = 0.40 [47]. Although significant effects were no longer present at the 6-month follow-up, change in (meta-)cognitive biases remained stable [45]. The nonsignificant effects at follow-up may potentially derive from the fact that the patients did not practice the exercises on a regular basis. Despite the generally positive subjective appraisal of the myMCT, 67 to 83% of patients mentioned that instead of using myMCT as a self-help treatment, they would like to use it in face-to-face psychotherapy [44, 45]. To meet this preference, we converted the myMCT to a group format that is comprised of four modules primarily targeting (meta-)cognitive biases and beliefs, and this was positively evaluated by patients in a pilot trial [48–50]. In the pilot trial patients with OCD participated in the MCT-OCD, which included four modules that were conducted over 4 weeks during their inpatient stay in a single-arm trial. Acceptability of the MCT-OCD was high; for example, 89.7% of the patients said they would recommend the MCT-OCD to others and thought the training was useful and understandable [48]. Furthermore, Miegel et al. [50] provided some evidence that the different modules indeed specifically improved targeted (meta-)cognitive biases and beliefs: for example, the subjective need to control thoughts was especially reduced after a module targeting control of thoughts. This indicates that the MCT-OCD modules specifically reduce the biases that are addressed in each module. Evaluation of the effectiveness of the MCT-OCD pilot study demonstrated that patients’ OC symptoms decreased with a large effect from baseline to post assessment (η^2^_partial_ = 0.50) [49]. However, the pilot study lacked a control group and included only patients currently undergoing a comprehensive inpatient treatment, limiting the conclusions.

The results of the pilot study served to revise the MCT-OCD, and this revised version will be evaluated in a randomized controlled trial with an outpatient sample in the proposed study, which may allow more robust conclusions about the efficacy of the intervention. The MCT-OCD in a group format comes with the general advantages of group therapy (e.g., patients are able to share their thoughts with others who have similar symptoms and obstacles), which has already been shown to be very helpful for patients with OCD for CBT groups [51].

### Specific contents of the MCT-OCD

The revised version of the MCT-OCD is comprised of eight modules (in contrast to the four modules of the pilot version) that aim at modifying patients’ dysfunctional (meta-)cognitive beliefs, biases as well as dysfunctional coping strategies. The modules suggest functional coping strategies by first introducing the concept of the respective dysfunctional process and subsequently providing new, more functional coping strategies for dealing with the various dysfunctional (meta-)cognitive beliefs, biases as well as dysfunctional coping strategies. Modules 2 to 7 target OCD-specific cognitive biases identified by the OCCWG [52–54]: perfectionism (module #2), intolerance of uncertainty (module #3), action fusion (module #4), control of thoughts (module #5), overestimation of threat (module #6), and inflated sense of responsibility (module #7). Module #1 provides general information about OC symptoms (obsessions and compulsions), their consequences (i.e., avoidance and safety behavior), as well as myths about OCD (e.g., OCD is exclusively genetically determined and cannot be treated). The last module (module #8) addresses two cognitive biases: biased attention and biased cognitive networks. In this module, the cognitive intervention known as association splitting [55–59] that our working group developed is introduced (see Table 1 for a detailed description of all modules).

**Table 1:** Detailed Description of All MCT-OCD Modules and Exercises

Description of all modules	Example exercises (homework)
**1. False assumptions about OCD**	1. Write down your personal obsessions, compulsions, and avoidance as well as safety behaviors and develop your own cognitive model for OCD.
False assumptions about OCD (e.g., OCD is very rare) are corrected, the cognitive model of OCD [60] is introduced, and alternative behavior strategies (e.g., asking their family not to react to their reassurance seeking) are suggested.	
	2. Compose a goodbye letter to your obsessions.
**2. Perfectionism**	1. Nobody’s perfect. Pay attention to the failures or imperfections of people you admire.
The advantages and disadvantages of doing something accurately as well as the right balance of accuracy and errors are discussed. Acceptance strategies are displayed in order to learn how to handle “imperfections.”	
	2. Deliberately be imperfect, observe the consequences, and write them down.
**3. Intolerance of uncertainty**	
Advantages and disadvantages of intolerance of uncertainty and the role of negative emotions during the experience of obsessions are discussed. The use of sentences that create a distance between an obsession and reality are suggested (e.g., “This is an obsessive thought, not reality”). Additional slides regarding depressive thought patterns are included that address, for example, overgeneralization (e.g., “I always do everything wrong”).	
	1. Find alternative evaluations of an incident where you were prone to overgeneralization.
	2. Write down your strengths as well as explicit situations where you displayed them.
**4. Action fusion**	1. Try to influence someone else’s actions, an object, or an incident with your thoughts and use a checklist to see if you were successful.
It is explained that everybody is sometimes prone to thought-action fusion. The role of emotions during the occurrence of thought-action fusion is discussed, a thought behavioral exercise is practiced, and the difference between (aggressive) thoughts and actions is highlighted.	
	2. Try to influence an incident only with your thoughts and write down what happens. (The goal is for the patients to learn that thoughts cannot influence actions, objects, or incidents.)
**5. Control of thoughts**	1. Try one of the imagination exercises presented in group (e.g., imagine clouds passing by) and write down what you experience.
The impossibility of completely controlling one’s thoughts is addressed (e.g., thought suppression). The vicious circle of aggression, guilt, and disappointment is explained, and patients are encouraged to let aversive thoughts pass by—like clouds, for example— in an imagination exercise.	
	2. Find sentences that help to create a distance from your obsessions (e.g., “This is an obsessive thought, not reality”).
**6. Overestimation of threat**	1. Write down your personal obsessional fear, the estimated possibility that it will occur, new information about your fear, alternative thoughts, and the converse probability.
Reasons for overestimation of threat are displayed (e.g., unrealistic pessimism). Calculating the statistical likelihood of a feared incident is practiced. Additional slides on rumination help patients to differentiate between rumination and normal problem-solving and provide a behavioral exercise that helps them to disengage from rumination.	
	2. Calculate the likelihood that your obsessional fear will occur.
**7. Inflated sense of responsibility**	1. Practice and write down your experiences while actively changing your perspective.
The relevance of an exaggerated sense of responsibility in OCD is highlighted. An active change of perspective is suggested. Patients practice finding more diverse reasons for particular events (i.e., others, coincidence, oneself) and are encouraged to counter attributing causations solely to themselves.	
	2. Write down three reasons for the occurrence of an event that fall into the categories “others,” “coincidence,” and “oneself” in order to counter attributing causation solely to oneself.
**8. Biased attention/biased cognitive networks**	1. Practice guiding your attention to a stimulus and write down what you experience.
Patients are encouraged to guide their attention purposely to certain stimuli in order to disengage from biased attention toward their feared stimuli. Patients learn how cognitions are associatively linked. The technique of “association splitting” is introduced in order for patients to form new associations and weaken obsessive ones.	
	2. Write down an OCD-relevant word and then write down new neutral or positive associations. Practice these associations for 10 min a day in order to weaken old OCD-relevant associations and form new ones.

While all beliefs dealt with in the revised version were also addressed in the pilot version of the MCT-OCD (two biases per module), general information on OCD, which is now provided in module #1 of the revised MCT-OCD, was not included in the pilot version. This information would have been redundant with other treatments of the inpatient sample. In order to ensure that patients are provided with all relevant basic information about OCD (e.g., information about false assumptions about OCD and more general information about obsessions, compulsions, and avoidance as well as safety behavior), this information is presented in module #1. Moreover, slides on additional topics were added in two modules that target depression and rumination because OCD and depression have a lifetime comorbidity of 56.6% [61] and share some dysfunctional beliefs and coping strategies, such as rumination [62–64].

### Aim of the present study

The present study aims to evaluate the efficacy of the MCT-OCD versus a care as usual control group (i.e., patients are allowed to continue their treatment as usual and/or start a new treatment) for patients with OCD. The MCT-OCD explicitly targets (meta-)cognitive beliefs, biases as well as dysfunctional coping strategies that are relevant and potentially specific to OCD and contribute to the development and maintenance of OC symptoms [52–54, 65]. We hypothesize that patients who participate in the MCT-OCD will display significantly lower symptom severity than patients in the care as usual control group at post assessment. In particular, we hypothesize that MCT-OCD will lead to a greater reduction in OC symptoms as measured by the Yale-Brown Obsessive Compulsive Scale (Y-BOCS; total score, primary outcome), compared to a usual care control group over a period of 8 weeks. We also hypothesize that symptom reduction will be maintained at follow-up assessment (3 months after post, secondary outcome). Additionally, as the MCT-OCD also targets cognitive biases suggested by the OCCWG [52–54] as well as beliefs related to depressive symptoms, which are also highly relevant for patients with OCD [62–64], we also hypothesize that the dysfunctional (meta-)cognitive beliefs, biases as well as the depressive symptoms will show a stronger decline while the quality of life will increase more strongly in the intervention group compared to the control group. Accordingly, we assume that the MCT-OCD will lead to a greater reduction (or increase with regard to quality of life) in beliefs as assessed by the Obsessive Beliefs Questionnaire (OBQ), symptoms of depression as measured by the Hamilton Depression Rating Scale (HDRS) and the Beck Depression Inventory-II (BDI-II), metacognitions as measured by the Metacognitions Questionnaire-30 (MCQ-30), quality of life as measured by the World Health Organization Quality of Life Assessment (WHOQOL-BREF), and the frequency and distress experienced due to OC symptoms assessed by the Obsessive Compulsive Inventory-Revised (OCI-R) compared to the care as usual control group over a period of 8 weeks (secondary outcomes). We expect a positive appraisal of the MCT-OCD similar to the pilot version of the MCT-OCD [48].

## Methods

### Study design and ethical aspects

The study is designed as an assessor-blind, randomized controlled trial with an intervention group (MCT-OCD) and a care as usual control group. The study is registered with the German Registry for Clinical Studies (DRKS00013539), was approved by the local ethics committee (Deutsche Gesellschaft für Psychologie; LJ112017), and will be conducted in accordance with the Declaration of Helsinki. All information revealing patient identity (name, e-mail address, etc.) will be stored separately from psychopathological data in a locked cabinet. A coding list will be created in which the names of the patients are linked to the corresponding identifiers. The coding list will only be available in paper form and will be kept in a locked cabinet. Once data collection is completed, the coding list will be destroyed. The anonymized data will be archived for 10 years. Written informed consent will be obtained from all participants. If participants withdraw their consent to participate in the study, the reasons will be carefully documented and the collected data will be destroyed and deleted. Only staff members directly involved in the project and approved by the principle investigator will have access to the final dataset.

### Sample size

To detect a medium to large effect size of *d* = 0.80, with an alpha level of α = 0.05 and a power of 0.80, G*Power [66] calculated a sample size of *N* = 70. As a dropout rate of 15% is expected, a sample of *N* = 80 patients with OCD will be recruited and randomized to the two groups (*n* = 40 MCT-OCD, *n* = 40 usual care control group). A diagnosis of OCD will be verified by the Mini International Neuropsychiatric Interview 5th Ed. (MINI 7.0.2) [67], which serves to elucidate further psychiatric diagnoses based on the *Diagnostic and Statistical Manual of Mental Disorders* (5th ed.) (*DSM-5*).

### Recruitment

Participants will be recruited via the anxiety outpatient clinic of the Clinic for Psychiatry and Psychotherapy of the University Medical Center Hamburg-Eppendorf (Germany), local therapists, Google AdWords, the German society for OCD (DGZ), posters, and brochures. Individuals who have already participated in prior studies of our working group and have given their written informed consent for future contact will also be contacted. Patients who already participated the MCT-OCD pilot version were not contacted again. Patients who read the myMCT were not excluded but this was carefully documented.

### In- and exclusion criteria

In order to be able to generalize the results to a broad population of OCD patients, the eligibility criteria for the recruitment of the sample have been chosen carefully. The following criteria have to be fulfilled to be included in the proposed study: Participants need to (a) be between 18 and 70 years of age; (b) have a diagnosis of OCD according to the MINI; (c) demonstrate the willingness to participate in the MCT-OCD training and provide informed consent; (d) be available to attend the weekly sessions, and (e) be suitable for group therapy. Nationality will be assessed during a telephone interview, and if the potential participant reports a non-German nationality, the interviewer will explore whether they have sufficient language comprehension. The patients’ suitability for participating in a group setting will be assessed during the screening verifying the social skills of the patients during the interview (e.g., whether patients can attend group rules, such as not to insult others). All participants receiving any kind of outpatient psychotherapy (e.g., CBT, CT, psychodynamic) and/or pharmacological treatment (e.g., SSRI, antidepressants) will be able to continue this treatment as usual. All treatments will be documented thoroughly throughout the study. Additional to the kind of treatment, the number of past treatments as well as the number of sessions of the current treatment will be assessed. The exclusion criteria are (a) current or lifetime psychotic symptoms (e.g., mania), (b) a severe neurological disease, (c) current substance dependence, and (d) current inpatient treatment.

### Procedure and randomization

Inclusion and exclusion criteria will be screened through interviews via telephone. If patients appear eligible, they will be invited for an in-person baseline interview and included after they have received detailed information on the study project and provided informed consent. Assessment will be conducted by trained research assistants (blind to group allocation) who have completed a rater training and have received individual feedback from experts in interviews beforehand. Participants are assessed at three timepoints: baseline (−t1), 8 weeks after baseline (t1), and 3 months after t1 (t2).

**Fig. 1:**
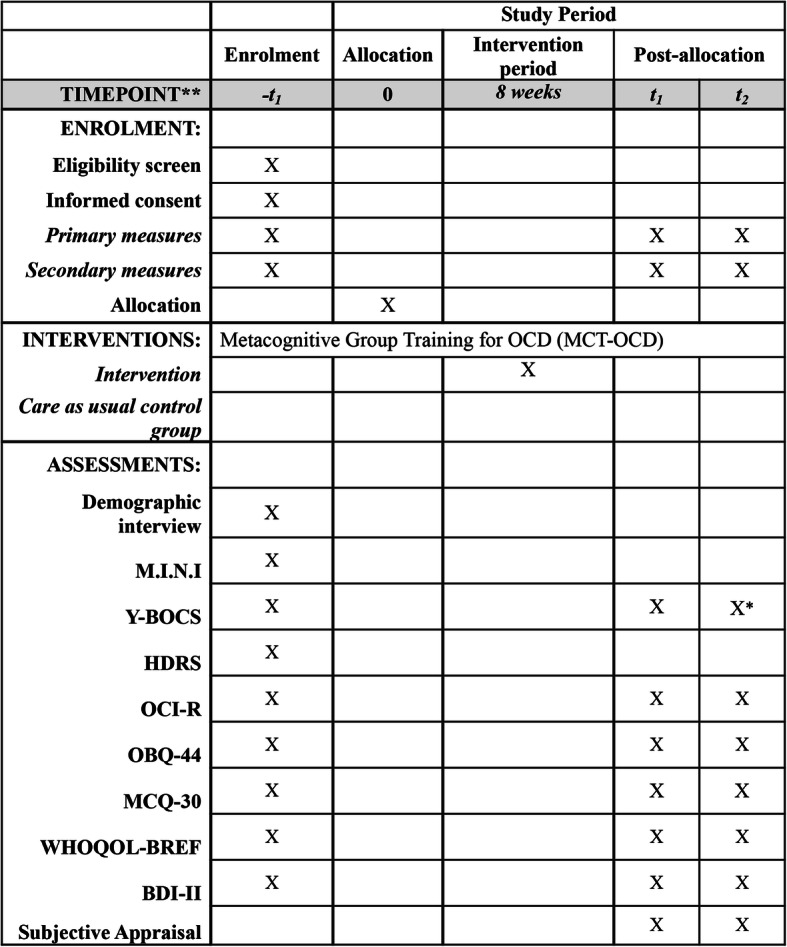
Standard protocol items: recommendation for interventional trials (SPIRIT) timeline. *Y-BOCS was administered as a self-rating

At the baseline interview, demographic information and the OC symptoms are recorded through self and expert ratings. The MINI 7.02 [67], a semi-structured interview, will be used to verify a diagnosis of OCD and to record comorbid mental disorders as well as to check for inclusion or exclusion criteria. Symptom severity will be measured using the Y-BOCS that will be administered in person at baseline and post assessment and assessed online at follow-up [68]. The german vocabulary test (Wortschatztest [69]) will be assessed as part of the baseline assessment, which can be used as an IQ estimator. In addition, patients will be asked to fill out self-report questionnaires. Due to the high comorbidity of OCD and depression [61], as well as the revisions made to the MCT-OCD pilot version (i.e., inclusion of slides that target depression and rumination) a comprehensive assessment of depressive symptoms is planned. We therefore used the Beck Depression Inventory-II (BDI-II) [88] and the Hamilton Depression Rating Scale (HDRS) [70], which focus on different facets of depression. For a detailed summary of all instruments, see Fig. 1.

### Randomization and assessor blindness

The randomization will be carried out via a computerized randomization plan, which will not be accessible to the assessors (blinded). Randomization will take place after baseline assessment (−t1): Patients will receive a prepared, sequentially numbered envelope with a letter stating the group they are allocated to (intervention group [MCT-OCD] or care as ususal control group) from the person coordinating the study. After the last MCT-OCD session (or 8 weeks after –t1 for the usual care control group), patients will be invited to participate in the post assessment (t1) in order to re-assess primary and secondary outcomes. Three months later, participants will receive a link to an online survey for the follow-up assessment (t2) via email. Subsequently, participants who were assigned to the care as ususal control group will be allowed to participate in the MCT-OCD to improve adherence. Withdrawal from the intervention or assessments will be possible at any time. Before the post assessment, patients will be reminded not to reveal their intervention condition to the assessor.

### Primary outcome measure

#### Yale-Brown Obsessive Compulsive Scale (Y-BOCS)

The Y-BOCS ([68]; German version: [71]) is a half-structured interview and is regarded as the gold standard for assessing OC symptom severity. The instrument is comprised of two parts: A symptom checklist to identify current as well as former OC symptoms, which identifies the three main obsessions and compulsions, and structured questions designed to determine symptom severity over the course of the past 7 days. In both parts, the main obsessions and compulsions are inquired separately. For the proposed study, the reduction in the Y-BOCS total score (the first ten items of the second part) from baseline to post assessment will be the primary outcome.

### Secondary outcome measures

#### Hamilton Depression Rating Scale (HDRS)

The Hamilton Depression Rating Scale (HDRS) [70], a semi-structured interview, is utilized to assess depressive symptom severity and frequency over the past 7 days. In the present study, the 17-item version of the HDRS will be used. The severity is rated on a scale ranging from one to five, with a maximum rating of 52. According to Kriston and von Wolff [72], a final rating of seven or less can be interpreted to mean the patient is not depressed. The HDRS is a widely used instrument and holds a good internal consistency of α = .79, an interrater reliability of *r* = .94, and a test-retest reliability of *r* = .87 [73].

#### Obsessive Compulsive Inventory (OCI-R)

The OCI-R ([74]; German version [75]) assesses the frequency and distress experienced due to OCD symptoms across six subscales: washing, obsessing, hoarding, ordering, checking, and neutralizing. The OCI-R contains good psychometric properties [74, 76, 77] that have been confirmed for the German version [75, 78]. It is sensitive to change [79]. Internet administration of the OCI-R produces equivalent results to paper-and-pencil administration [80].

#### Obsessive Beliefs Questionnaire (OBQ-44)

The OBQ-44 ([81, 82]; German version [83]) is a 44-item self-report questionnaire targeting beliefs in OCD on six subscales: control of thoughts, importance of thoughts, responsibility, intolerance of uncertainty, overestimation of threat, and perfectionism. It shows good psychometric properties with a high internal consistency [54, 82] and good convergent and discriminant validity [54].

#### Metacognitions Questionnaire (MCQ-30)

To assess dysfunctional metacognitive beliefs according to Wells’ model, the 30-item MCQ-30 [84] is used. The questionnaire assesses five subscales: cognitive confidence, positive beliefs about worry, cognitive self-consciousness, negative beliefs about the uncontrollability of thoughts and danger, and beliefs about the need to control thoughts. The MCQ-30 demonstrates high internal consistency (Cronbach’s α = .72 to .93) [84] and has been shown to have good convergent validity [85].

#### Quality of life (WHOQOL-BREF)

The WHOQOL-BREF [86], a 26-item short form of the WHOQOL-100, is a valid and reliable instrument for assessing quality of life [87]. In the current study, we will only use the global item of the WHOQOL-BREF (How would you rate your quality of life?), which has to be answered on a 5-point Likert scale (*very poor* to *very good*).

#### Beck-Depression Inventory (BDI-II)

The Beck Depression Inventory-II [88] contains 21 items assessing cognitive, behavioral, and somatic symptoms of depression over the past 2 weeks. Items are answered on a 4-point Likert scale resulting in total scores ranging from 0 to 63 (0–8 no depression, 9–13 minimal depression, 14–19 mild depression, 20–28 moderate depression, and 29–63 severe depression). The German version of the BDI-II shows good psychometric properties in clinical and nonclinical samples [89].

#### Subjective appraisal rating of the MCT-OCD

A 21-item rating scale to assess the subjective appraisal of the MCT-OCD will be used. A similar questionnaire has been used for the evaluation of the D-MCT [90] and the pilot version of the MCT-OCD [48]. The items range on a 5-point Likert scale from 1 = *totally agree* to 5 = *totally disagree*, and two open questions ask participants to appraise the MCT-OCD. This questionnaire will be administered at the post and follow-up assessments.

### Intervention

The MCT-OCD aims to modify dysfunctional (meta-)cognitive biases, beliefs as well as dysfunctional coping strategies that contribute to the development and maintenance of OC symptoms. The number of modules has been increased from four (pilot version) to eight (revised version) and the length of the sessions extended from 60 to 90 min (over the period of 8 weeks) in order to provide more time for the presentation of the content as well as for addressing the concerns of patients. Two persons who do not have much experience in conducting group therapies and have not completed a training course (one a psychologist undergoing post-graduate training and the other an assisting intern with a bachelor’s degree) will conduct the sessions. Three to ten patients will take part in each session. As the MCT-OCD has an open group format, patients can join the group at any time. At the beginning of their first session, patients will receive a booklet that includes a summary as well as exercises for each module. Six of the eight modules deal with one dysfunctional (meta-)cognitive belief, bias or coping strategy at a time and follow the same structure: (1) an explanation of the general idea of the metacognitive training (only if new participants join the group), (2) a discussion of the exercises from the previous session, (3) an introduction of the dysfunctional (meta-)cognitive belief, bias or dysfunctional coping strategy, (4) examples and emphasis of the relevance for OCD, (5) a presentation and the practicing of techniques to overcome these (meta-)cognitive biases, beliefs or coping strategies, (6) a recap of the main content of the present module, and (7) the possibility for patients to ask questions and to say what they found most helpful. One exception is module #1 (false assumptions about OCD), which teaches patients very basic information about OCD by clarifying false assumption about the disorder. The second exception is module #8 (biased attention/biased cognitive networks), which addresses two cognitive biases (both are relatively short), but the structure is the same as in the other modules. Modules #3 and #6 include additional slides on how to deal with depression and rumination because OCD and depression have a very high comorbidity [61]. If a participant reports negative effects from the MCT-OCD, the therapist will assist the patient in finding another treatment.

### Statistical analyses

An intention-to-treat analysis (considering patients who provide baseline data) as well as complete cases analysis (CC; considering patients who provide baseline, post, and/or follow-up data) will be performed. ANCOVAs with treatment as the between-subject factor (MCT-OCD vs. care as usual control group), the baseline level of the respective outcome as the covariate, and the difference in the scores of the outcomes (t1 – (−t1) and t2 – (−t1), respectively) as the dependent variable will be conducted [91]. Differences between the groups at baseline (−t1) will be analysed by an independent samples *t*-test (for continuous variables) or a chi-square test (for categorical variables). For the intention-to-treat analyses, multiple imputation will be used for missing values. The number of patients that attained response and/or remission will be reported following the suggestions by Mataix-Cols et al. [21]. Additional regression analyses will be conducted in order to identify variables (severity of baseline OCD symptoms, baseline depression, number of sessions completed, and type of OCD symptoms [e.g., washing, checking], prior therapy experiences etc.) that contribute to the treatment effectiveness of the MCT-OCD.

## Discussion

The present study is the first to investigate the efficacy of the revised Metacognitive Training for patients with OCD (MCT-OCD) in the framework of a randomized controlled trial. The MCT-OCD has two major strengths: It (1) allows for easier dissemination (e.g., in comparison to CBT) and (2) provides a well-accepted treatment for patients with OCD. Easy dissemination may be achieved due to the MCT-OCD’s open group format, its high standardization due to the slide-supported presentation, and its potential to be conducted by therapists and other health care personell without advanced professional training. Furthermore, the MCT-OCD contains elements of CT, and is a comprehensive program. It is “rooted in the setup and presentation mode of the Metacognitive Training for Psychosis, [which] disorder-specific versions have been intended as hybrids, to amalgamate a CBT and Metacognitive Training approach” ([15], p. 5). Thus, a potential efficacy of the MCT-OCD cannot solely be attributed to the metacognitive elements. Moreover, the MCT-OCD has the advantage of targeting accompanying depressive beliefs and symptoms along with OCD. The importance of targeting dysfunctional (meta-)cognitive beliefs, biases as well as dysfunctional coping strategies in the treatment of OCD has already been demonstrated [6–8, 10–12].

Our working group cooperates with the anxiety outpatient clinic of the Clinic for Psychiatry and Psychotherapy of the University Medical Center Hamburg-Eppendorf, as well as with the German Society for OCD (DGZ). Moreover, our previous projects indicate that recruitment via a local Google AdWords campaign can be very successful, so the recruitment of a sample size of 80 patients is deemed realistic within the study period. To improve completion rates, patients in the usual care control group will be offered the opportunity to participate in the MCT-OCD after they have filled out the online questionnaire 5 months after the baseline assessment. Patients in both groups will receive questionnaires and a reminder of the upcoming meeting 1 week before t1. Moreover, a short 3-month interval between post and follow-up assessments was chosen to minimize dropout after post assessment.

The trial has some limitations that need to be acknowledged. First, we assume that the MCT-OCD group will receive a larger amount of professional attention (i.e., 90 min per week) than the control group during the intervention period (regardless of the content). Thus, therapeutic alliance might have an impact on the results [92]. Second, care as usual control groups in contrast to active control groups come with the disadvantage of not being able to eliminate expectancy-effects [93]. However, active control treatments are costly and a first important step is most commonly to compare a treatment to care as usual (or even a no-treatment waiting control group). Third, at follow-up assessment the trial relies on the self-rating of the Y-BOCS. Self-ratings come with several disadvantages (e.g., social desirability) [94]. However, several studies support the validity of the Y-BOCS self-rating [96] and was chosen after a cost-benefit consideration. Forth, as an 8-week period of time between baseline and post assessment may produce a higher dropout rate, an additional assessment after 4 weeks would be desirable if more resources are available. But as described above we are confident to be able to reach a high completion rate.

One of the strengths of the revised MCT-OCD, in our view, is that it treats depressive symptoms in addition to dysfunctional (meta-)cognitive beliefs, biases and dysfunctional coping strategies. OCD and depression have a high comorbidity [61], thus depressive symptoms, especially rumination [62–64], are highly relevant as targets for patients with OCD. Other strengths of the study’s design include the large sample size and the assessor-blinded randomization as well as the comprehensive test battery.

Besides showing promising in-session as well as between-session effects, the MCT-OCD in its pilot version has also been shown to be highly accepted by patients with OCD [48], to have module-specific positive effects [50], and to result in a reduction of OC symptoms with large effect sizes [49]. As OCD patients often do not receive the most effective treatment for OCD (CBT with ERP), the MCT-OCD aims to provide a treatment option that is low threshold and highly accepted by patients. If proven effective against care as usual, it may help reduce the burden of OCD, as the MCT-OCD is highly standardized and easy to administer and can therefore be integrated quickly and economically into everyday clinical practice. As part of a larger stepped-care approach, MCT-OCD could, for example, be used as a sole intervention for mild cases or to bridge waiting times and ease the start of treatment with ERP.

### Trial status

The first participant was enrolled in February 2018. At the time of submission of this study protocol, protocol participants were still being recruited and no data had yet been analyzed. Any future changes to the study protocol will be recorded in a separate amendment. SPIRIT guidelines were followed for the entire manuscript.

## Data Availability

Not applicable.

## Abbreviations

CBT: Cognitive behavioral therapy
CT: Cognitive therapy
ERP: Exposure and response prevention
myMCT: My metacognitive training
MCT: Metacognitive training
MCT-OCD: Metacognitive training for patients with OCD
OCD: Obsessive compulsive disorder
OC: Obsessive compulsive
OCCWG: Obsessive compulsive cognitions working group
TAF: Thought-action fusion

## Footnotes

1 Metacognition is defined as thinking about thinking or as cognition about cognitive processes [14].
